# Clinical-Deep Neural Network and Clinical-Radiomics Nomograms for Predicting the Intraoperative Massive Blood Loss of Pelvic and Sacral Tumors

**DOI:** 10.3389/fonc.2021.752672

**Published:** 2021-10-25

**Authors:** Ping Yin, Chao Sun, Sicong Wang, Lei Chen, Nan Hong

**Affiliations:** ^1^ Department of Radiology, Peking University People’s Hospital, Beijing, China; ^2^ Department of Pharmaceuticals Diagnosis, GE Healthcare (China), Shanghai, China

**Keywords:** deep neural network, radiomics, pelvic tumors, blood loss, computed tomography

## Abstract

**Background:**

Patients with pelvic and sacral tumors are prone to massive blood loss (MBL) during surgery, which may endanger their lives.

**Purposes:**

This study aimed to determine the feasibility of using deep neural network (DNN) and radiomics nomogram (RN) based on 3D computed tomography (CT) features and clinical characteristics to predict the intraoperative MBL of pelvic and sacral tumors.

**Materials and Methods:**

This single-center retrospective analysis included 810 patients with pelvic and sacral tumors. 1316 CT and CT enhanced radiomics features were extracted. RN1 and RN2 were constructed by random grouping and time node grouping, respectively. The DNN models were constructed for comparison with RN. Clinical factors associated with the MBL were also evaluated. The area under the receiver operating characteristic curve (AUC) and accuracy (ACC) were used to evaluate different models.

**Results:**

Radscore, tumor type, tumor location, and sex were significant predictors of the MBL of pelvic and sacral tumors (*P* < 0.05), of which radscore (OR, ranging from 2.109 to 4.706, *P* < 0.001) was the most important. The clinical-DNN and clinical-RN performed better than DNN and RN. The best-performing clinical-DNN model based on CT features exhibited an AUC of 0.92 and an ACC of 0.97 in the training set, and an AUC of 0.92 and an ACC of 0.75 in the validation set.

**Conclusions:**

The clinical-DNN and clinical-RN had good performance in predicting the MBL of pelvic and sacral tumors, which could be used for clinical decision-making.

## Introduction

Pelvic and sacral tumors have various types, among which metastatic tumors are the most common. Chondrosarcoma is the most common primary malignant bone tumor that occurs in the pelvis, followed by osteosarcoma and Ewing’s sarcoma (1, 2). Sacral chordoma and giant cell tumors are the two most common primary sacral tumors (3). Given the complex anatomical structure and large volume of pelvic and sacral tumors, their surgical resection is a challenging procedure and can be complicated by massive blood loss (MBL).

The prediction of intraoperative blood loss is an important component of preoperative planning, and an accurate assessment will facilitate intraoperative and postoperative management (4). A limited number of previous studies with small sample sizes have analyzed the factors that affect the amount of blood loss in spinal tumors (5–9). Tang et al. (10) retrospectively reviewed 173 patients who underwent sacral tumor resection and found that tumors invading the cephalad to the S2–S3 disc space with a volume greater than 200 cm^3^ and an abundant blood supply are likely to have a large amount of blood loss. Preoperative embolization and aortic balloon occlusion have been shown to reduce intraoperative blood loss in pelvic and sacral tumors and allow for a more complete resection (11–13). Nevertheless, the estimation of intraoperative blood loss is usually based on a surgeon’s personal experience in clinical practice. Misjudgment in preoperative evaluation may endanger the patient’s life or cause the waste of blood products (7). Therefore, establishing a prediction model might more adequately lower intraoperative MBL than subjective experience alone and may therefore improve patient outcomes.

The recent advances and future perspectives of machine learning techniques provide promising applications for medical imaging (14). Radiomics is a subfield of machine learning dedicated to extracting quantitative features from radiological images by using specific algorithms that allow obtaining information beyond conventional medical imaging analysis (15, 16). The deep learning algorithm has been widely used in the field of image diagnosis and prediction due to its advantages of rapidity, accuracy, and good reproducibility (17–19). Ryu et al. (20) developed deep neural network (DNN) machine learning algorithms to predict survival following diagnosis with spino–pelvic chondrosarcoma. However, their model was solely based on clinical data without considering imaging features. Yin et al. (21) built a clinical-radiomics nomogram (RN) combining computed tomography (CT) features with clinical data. They found that clinical-RN performs better than the individual clinical model for the differentiation of sacral tumors. Although DNN and radiomics have been used in tumor diagnosis, efficacy evaluation and prognosis prediction in recent years, their application in the prediction of intraoperative blood loss in pelvic and sacral tumors has not been reported (22).

The aim of our study was to investigate the feasibility of using DNN and radiomics approaches based on CT features and clinical characteristics to predict the intraoperative MBL of pelvic and sacral tumors.

## Materials and Methods

### Patients

**Figure 1:**
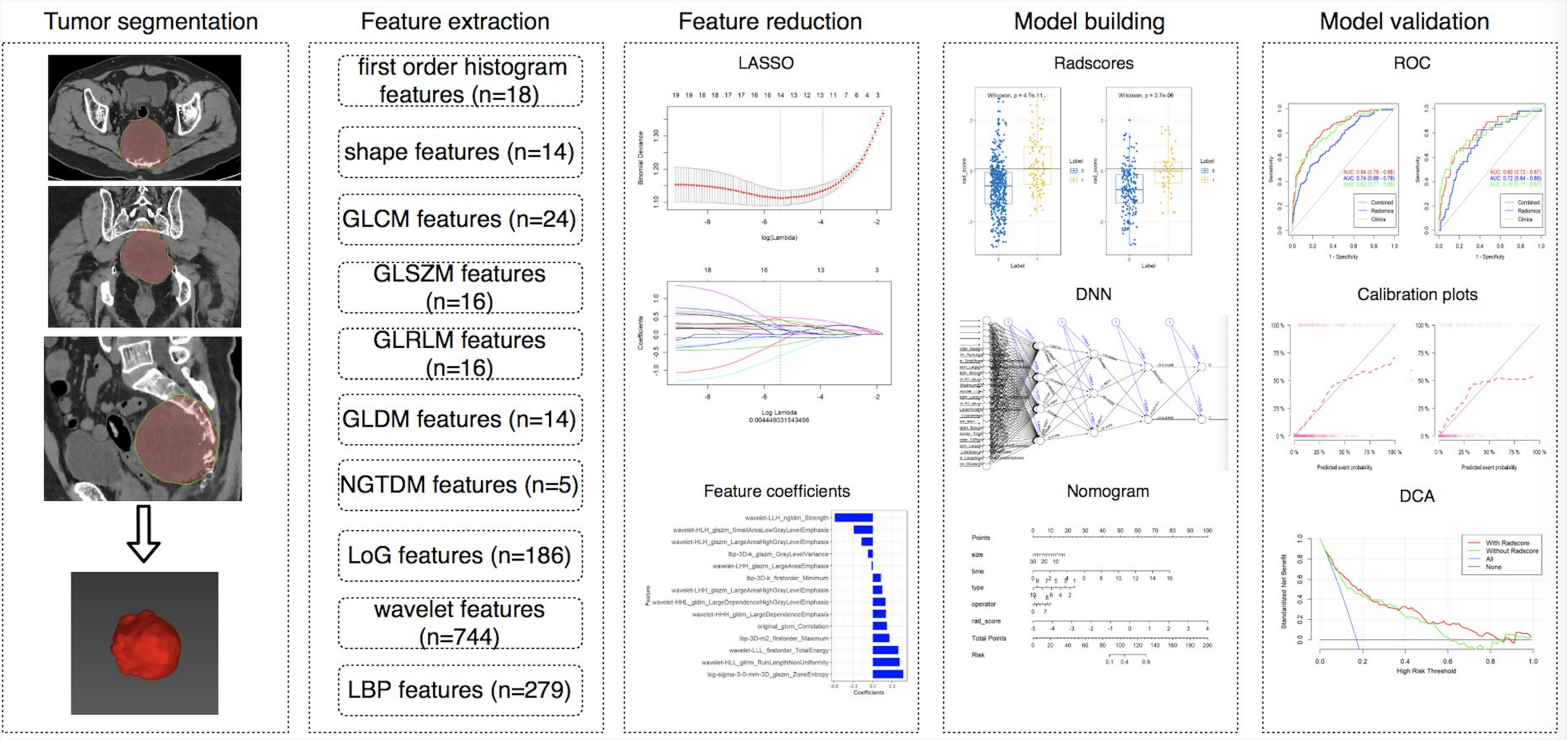
The workflow of this study.

This single-center retrospective study was approved by the local ethics committee of our hospital, and written informed consent was waived. A total of 1010 patients with pathologically confirmed pelvic and sacral tumors who underwent surgery in our institution from July 2005 to December 2019 were retrospectively analyzed. The inclusion criteria were as follows: (1) tumors were found on CT performed within 1 month before the first surgery; (2) preoperative CT or CT enhanced (CTE) images were complete and of good quality; and (3) pathology reports confirmed pelvic and sacral tumors. Patients without preoperative CT images (n = 84), with obvious artifacts (n = 10), or without surgery (n = 106) were excluded. Finally, a total of 810 patients were included in the study. However, 167 patients did not receive enhancement scans. Thus, we analyzed the CT data of 810 patients and CTE data of 643 patients, respectively. **Figure 1** shows the workflow of this study.

### Risk Factors

The following risk factors that were potentially associated with MBL were analyzed: sex, age, maximal tumor size, tumor type (chondrosarcoma, osteosarcoma, chordoma, giant cell tumor, multiple myeloma, schwannoma, neurofibroma, Ewing’s sarcoma, metastatic tumor, and others), tumor location (zone I–IV) (23), neoadjuvant chemo-radiotherapy, surgical methods, surgical approaches (posterior, combined anterior and posterior), surgeon, preoperative embolization, and aortic balloon occlusion (10, 11, 13, 24). Surgical methods included the resection of left or right pelvic tumors plus artificial hemipeleal replacement, the curetomy/resection and internal fixation of sacral tumors, and the resection and internal fixation of sacral and pelvic tumors. All the operations were performed by skilled surgeons with more than 10 years of experience in pelvic and sacral tumor surgery. Intraoperative blood loss was estimated by surgeons and anesthesiologists by measuring suction loss and weighing wound swabs. A blood loss of more than 3000 mL is considered to be massive (10).

### CT Acquisition

The details of this part are described in Electronic **Supplementary Material**.

### Tumor Segmentation

MITK software version 2018.04.2 (www.mitk.org) was used for manual segmentation (25). A semi-automatic delineation method was used for all lesions. We first manually delineated the edge of the lesion at the axial, sagittal, and coronal sites, and the software automatically formed a three-dimensional lesion, which was then manually corrected by a musculoskeletal radiologist with 5 years of experience and a senior musculoskeletal radiologist with 20 years of experience.

### Feature Extraction and Reduction

The details of this part are described in Electronic **Supplementary Material**.

### Model Building and Validation

Considering the imbalance between the groups, we used two methods to group data. First, we randomly divided the training group and the validation group in accordance with the ratio of 7:3, and used the synthetic minority oversampling technique (SMOTE) algorithm on the training set to amplify the data and reduce data imbalance (26). We built the first model (RN1), which was based on individual CT and CTE features, by using logistic regression (LR). Then, we divided the data into the training group and the validation group in accordance with the time node and built the second model (RN2) based on individual CT and CTE features. This method was used to divide the data from July 2005 to December 2016 into the training group and the data from January 2017 to December 2019 into the validation group. For CT features, the training group included 80 MBL cases and 320 non-MBL cases, and the validation group included 63 MBL cases and 347 non-MBL cases. For CTE features, the training group included 59 MBL cases and 245 non-MBL cases, and the validation group included 55 MBL cases and 284 non-MBL cases. Considering that LR performs best when the case-to-noncase ratio is 1:1 (27), we designed the training group to contain 80 patients with MBL and 80 patients without MBL who were randomly selected from the pool of 320 patients without MBL for CT features. Similarly, we also selected 59 patients with MBL and 59 patients without MBL from the training group for CTE features. We allocated all patients in the validation group to test our nomogram in a real experiment. The radiomics score (radscore) for each patient was calculated *via* a linear combination of selected features that were weighted by their respective coefficients. Then, we also built a DNN model based on selected features with a hidden layer number of 3. The number of hidden layer nodes in each layer is 4, 3, and 2, respectively (see **Supplemental Figure 1**).

Clinical risk factors were compared *via* univariate analysis, and variables with *P* value < 0.1 were included in the clinical model. Models were trained with the training set by using the repeated 10-fold cross-validation method, and estimation performance was evaluated with the validation set. When combined with clinical data, we also constructed the clinical-RN1, clinical-RN2, and clinical-DNN models.

### Statistical Analysis

The statistical analysis is reported in Electronic **Supplementary Material**.

## Results

### Patient Characteristics

A total of 810 patients (445 males, 365 females; mean age of 42.5 ± 17.3 years, range 4–85 years) were included in this study (**Table 1**). The overall blood loss ranged from 50 mL to 11000 mL with a median of 1500 (800, 2387.5) mL. The median of blood loss of all patients with MBL was 3900 (3220, 4920) mL, which was significantly higher than that of patients without MBL (1200 [800, 1780] mL) (*Z* = −18.78, *P* < 0.001). In the non-MBL group, 191 patients (28.64%) received neoadjuvant chemoradiotherapy, while only 38 patients (26.57%) received neoadjuvant chemoradiotherapy in the MBL group. No significant difference in intraoperative blood loss was observed between patients with embolism (1500 [900, 2312.5] mL) and those without embolism (1500 [800, 2400] mL) (*Z* = 0.937, *P* > 0.05). Patients with aortic balloon occlusion (1500 [900, 2500] mL) had significantly more blood loss than patients without occlusion (1200 [700, 2000] mL) (*Z* = 3.369, *P* < 0.05). In the CT group, MBL was found in 143 patients (17.7%), of which chondrosarcoma patients accounted for the largest proportion (25.9%). A similar result was found for the CTE group: 114 patients (17.7%) had MBL, with chondrosarcoma patients accounting for the highest proportion (26.3%). Univariate analyses showed that tumor type, tumor size, tumor location, operator, and operation method were significantly associated with MBL (*P* < 0.001). Chondrosarcoma and osteosarcoma tumor type, great tumor size, multiple locations, surgeons with low surgical experience, resection and internal fixation of sacral and pelvic tumors were likely to occur with MBL. No significant difference was found in terms of sex, age, neoadjuvant chemoradiotherapy, embolism, surgical approaches, and balloon occlusion between groups (*P* > 0.05).

**Table 1:** Clinical characteristics of patients.

Variable	CT		CTE		*χ^2^/Z* value	*P* value
	Non-MBL	MBL	Non-MBL	MBL		
Sex						
Female	311 (46.63%)	54 (37.76%)	245 (46.31%)	45 (39.47%)	3.738^a^ (1.772^b^)	0.053^a^ (0.183^b^)
Male	356 (53.37%)	89 (62.24%)	284 (53.69%)	69 (60.53%)		
Age (years)	44.0 (28.0, 56.0)	44.0 (27.0, 56.8)	44.0 (29.0, 56.0)	44.0 (27.0, 53.1)	0.022^a^ (0.648^b^)	0.983^a^ (0.517^b^)
Tumor type						
Metastatic tumor	118 (17.69%)	20 (13.99%)	89 (16.82%)	16 (14.04%)	45.343^a^ (42.202^b^)	<0.001^a,b^
Chordoma	68 (10.19%)	16 (11.19%)	57 (10.78%)	14 (12.28%)		
Giant cell tumor	94 (14.09%)	21 (14.69%)	74 (13.99%)	17 (14.91%)		
Osteosarcoma	68 (10.19%)	30 (20.98%)	57 (10.78%)	26 (22.81%)		
Chondrosarcoma	86 (12.89%)	37 (25.87%)	70 (13.23%)	30 (26.32%)		
Schwannoma	47 (7.05%)	2 (1.40%)	34 (6.43%)	2 (1.75%)		
Neurofibroma	43 (6.45%)	2 (1.40%)	35 (6.62%)	2 (1.75%)		
Ewing’s sarcoma	52 (7.80%)	8 (5.59%)	39 (7.37%)	5 (4.39%)		
Multiple myeloma	17 (2.55%)	1 (0.70%)	10 (1.89%)	0 (0.00%)		
Others	74 (11.09%)	6 (4.20%)	64 (12.10%)	2 (1.75%)		
Tumor size (cm)	8.5 (6.5, 11.3)	11.5 (8.8, 14.4)	8.6 (6.6, 11.2)	11.5 (9.0, 14.6)	-6.934^a^ (-6.633^b^)	<0.001^a,b^
Tumor location						
I	107 (16.04%)	16 (11.19%)	85 (16.07%)	13 (11.40%)	21.193^a^ (22.425^b^)	<0.001^a,b^
II	44 (6.60%)	9 (6.29%)	36 (6.81%)	5 (4.39%)		
III	44 (6.60%)	5 (3.50%)	34 (6.43%)	3 (2.63%)		
IV	351 (52.62%)	63 (44.06%)	271 (51.23%)	48 (42.11%)		
Multiple locations	121 (18.14%)	50 (34.97%)	103 (19.47%)	45 (39.47%)		
Neoadjuvant chemoradiotherapy						
No	476 (71.36%)	105 (73.43%)	376 (71.08%)	82 (71.93%)	0.247^a^ (0.033^b^)	0.619^a^ (0.855^b^)
Yes	191 (28.64%)	38 (26.57%)	153 (28.92%)	32 (28.07%)		
Embolism						
No	369 (55.32%)	77 (53.85%)	295 (55.77%)	67 (58.77%)	0.104^a^ (0.345^b^)	0.747^a^ (0.557^b^)
Yes	298 (44.68%)	66 (46.15%)	234 (44.23%)	47 (41.23%)		
Surgeon						
Surgeon 1	45 (6.75%)	5 (3.50%)	38 (7.18%)	4 (3.51%)	43.984^a^ (35.152^b^)	<0.001^a,b^
Surgeon 2	299 (44.83%)	53 (37.06%)	241 (45.56%)	44 (38.60%)		
Surgeon 3	66 (9.90%)	4 (2.80%)	55 (10.40%)	4 (3.51%)		
Surgeon 4	85 (12.74%)	24 (16.78%)	70 (13.23%)	17 (14.91%)		
Surgeon 5	44 (6.60%)	19 (13.29%)	30 (5.67%)	12 (10.53%)		
Surgeon 6	9 (1.35%)	6 (4.20%)	7 (1.32%)	5 (4.39%)		
Surgeon 7	13 (1.95%)	2 (1.40%)	10 (1.89%)	2 (1.75%)		
Surgeon 8	15 (2.25%)	1 (0.70%)	12 (2.27%)	1 (0.88%)		
Surgeon 9	33 (4.95%)	21 (14.69%)	27 (5.10%)	19 (16.67%)		
Surgeon 10	43 (6.45%)	5 (3.50%)	27 (5.10%)	4 (3.51%)		
Surgeon 11	15 (2.25%)	3 (2.10%)	12 (2.27%)	2 (1.75%)		
Operation methods						
Method1	365 (54.72%)	68 (47.55%)	285 (53.88%)	52 (45.61%)	28.825^a^ (22.733^b^)	<0.001^a,b^
Methods2	285 (42.73%)	57 (39.86%)	229 (43.29%)	47 (41.23%)		
Methods3	17 (2.55%)	18 (12.59%)	15 (2.84%)	15 (13.16%)		
Surgical approaches						
Approach1	634 (95.05%)	130 (90.91%)	499 (94.33%)	102 (89.47%)	3.774^a^ (3.621^b^)	0.052^a^ (0.057^b^)
Approach2	33 (4.95%)	13 (9.09%)	30 (5.67%)	12 (10.53%)		
Balloon occlusion						
No	178 (26.69%)	27 (18.88%)	134 (25.33%)	20 (17.54%)	3.795^a^ (3.122^b^)	0.051^a^ (0.077^b^)
Yes	489 (73.31%)	116 (81.12%)	395 (74.67%)	94 (82.46%)		

Operation methods, Method1 = resection of sacral tumors and internal fixation, Methods2 = resection of left or right pelvic tumors plus artificial hemipeleal replacement, Methods3 = resection and internal fixation of sacral and pelvic tumors. Approaches, Approach1 = posterior, Approach2 = combined anterior and posterior. a, CT. b, CTE.

### Performance of Different Models

In the randomization method, no significant statistical difference was observed between the training group and the validation group (*P* > 0.05) (see **Supplemental Table 1**).

**Figure 2:**
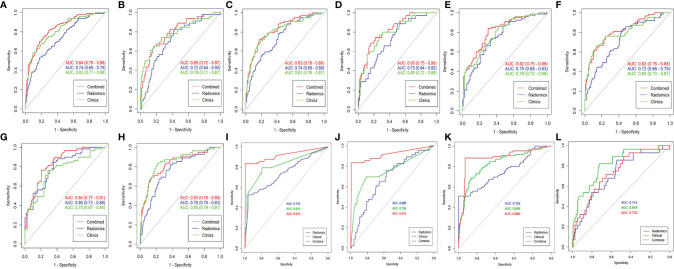
The ROC curve of different models. **(A, B)** CT-based RN1; **(C, D)** CTE-based RN1; **(E, F)** CT-based RN2; **(G, H)** CTE-based RN2; **(I, J)** CT-based DNN; **(K, L)**, CTE-based DNN. Column 1, 3, 5 is the training set, and column 2, 4, 6 is the validation set. The best-performing clinical-DNN based on CT features reached an AUC of 0.915 in both training set and validation set **(I, J)**.

In terms of CT features, the AUC of the validation set of RN1 and RN2 was 0.72, but a relatively higher ACC was found for the training and validation sets of RN1 (**Figure 2** and **Table 2**). Clinical-RN1 (AUC = 0.80, ACC = 0.80) performed better than RN1 (AUC = 0.72, ACC = 0.77) in the validation set. Also, an AUC of 0.82 and an ACC of 0.77 were found for clinical-RN2 in the validation set. The AUC and ACC of clinical-RN2 were higher than those of RN2 (AUC = 0.72, ACC = 0.61). DNN achieved a lower AUC of 0.68 and a higher ACC of 0.78 than RN1 and RN2 in the validation set.

**Table 2:** Performance of different models in training set and validation set.

	AUC	ACC	Sensitivity	Specificity	PPV	NPV
CT						
RN1	0.74 (0.72)	0.77 (0.77)	0.54 (0.41)	0.82 (0.85)	0.38 (0.40)	0.90 (0.86)
Clinics1	0.82 (0.79)	0.85 (0.84)	0.57 (0.61)	0.91 (0.89)	0.58 (0.50)	0.91 (0.92)
Clinical-RN1	0.84 (0.80)	0.80 (0.80)	0.45 (0.48)	0.93 (0.91)	0.70 (0.65)	0.83 (0.83)
RN2	0.75 (0.72)	0.70 (0.61)	0.74 (0.78)	0.66 (0.58)	0.69 (0.25)	0.72 (0.93)
Clinics2	0.79 (0.80)	0.72 (0.51)	0.67 (0.22)	0.81 (0.94)	0.86 (0.86)	0.58 (0.44)
Clinical-RN2	0.82 (0.82)	0.77 (0.77)	0.74 (0.36)	0.81 (0.95)	0.84 (0.76)	0.70 (0.76)
DNN	0.74 (0.68)	0.90 (0.78)	0.81 (0.32)	0.91 (0.82)	0.53 (0.15)	0.97 (0.92)
Clinics3	0.82 (0.76)	0.89 (0.84)	0.74 (0.61)	0.91 (0.89)	0.56 (0.50)	0.96 (0.92)
Clinical-DNN	0.92 (0.92)	0.97 (0.75)	0.96 (0.30)	0.97 (0.83)	0.84 (0.24)	0.99 (0.87)
CTE						
RN1	0.74 (0.73)	0.62 (0.61)	0.78 (0.71)	0.58 (0.58)	0.29 (0.27)	0.93 (0.90)
Clinics1	0.81 (0.80)	0.76 (0.74)	0.41 (0.38)	0.94 (0.92)	0.76 (0.71)	0.76 (0.74)
Clinical-RN1	0.83 (0.83)	0.81 (0.78)	0.47 (0.43)	0.93 (0.93)	0.72 (0.74)	0.83 (0.78)
RN2	0.80 (0.76)	0.75 (0.49)	0.85 (0.89)	0.64 (0.42)	0.70 (0.23)	0.81 (0.95)
Clinics2	0.75 (0.85)	0.74 (0.72)	0.73 (0.36)	0.75 (0.97)	0.76 (0.87)	0.71 (0.69)
Clinical-RN2	0.84 (0.83)	0.78 (0.83)	0.79 (0.49)	0.77 (0.93)	0.76 (0.69)	0.80 (0.86)
DNN	0.75 (0.71)	0.90 (0.82)	0.98 (0.32)	0.90 (0.87)	0.51 (0.21)	0.99 (0.92)
Clinics3	0.83 (0.82)	0.85 (0.88)	0.84 (0.83)	0.85 (0.88)	0.24 (0.18)	0.99 (0.99)
Clinical-DNN	0.89 (0.74)	0.92 (0.77)	0.75 (0.30)	0.97 (0.90)	0.88 (0.46)	0.93 (0.82)

RN, radiomics nomogram; AUC, area under curve; ACC, accuracy; PPV, positive predictive value; NPV, negative predictive value. Training set, in front of the brackets. Validation set, in brackets.

For CTE features, RN2 achieved a higher AUC (AUC_training_ = 0.80, AUC_validation_ = 0.76) than RN1 in the training and validation sets. The AUC of clinical-RN1 and clinical-RN2 was 0.83 in the validation set. However, the ACC value (0.83) of clinical-RN2 was slightly higher than that of clinical-RN1. Similarly, the AUC of DNN (AUC_training_ = 0.75, AUC_validation_ = 0.71) was relatively lower than that of RN1 and RN2, but the ACC (ACC_training_ = 0.90, ACC_validation_ = 0.82) improved.

The clinical model had a good performance in the training (AUC, ranging from 0.75 to 0.83) and validation sets (AUC, ranging from 0.76 to 0.85). When combined with clinical features, clinical-RN performed better than individual RN in terms of CT or CTE features. The clinical-DNN model based on CTE features performed well (AUC = 0.89, ACC = 0.92) in the training set but had a lower value (AUC = 0.74, ACC = 0.77) than the clinical model (AUC = 0.82, ACC = 0.88) in the validation set. The best-performing clinical-DNN model based on CT features exhibited an AUC of 0.92 and an ACC of 0.97 in the training set, and an AUC of 0.92 and an ACC of 0.75 in the validation set.

### Performance of Different Clinical-RNs

**Figure 3:**
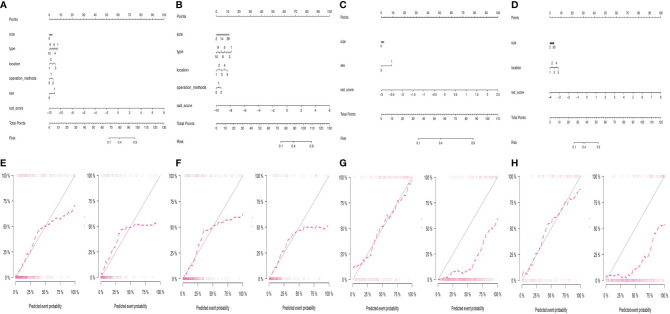
Performance of different clinical-RNs. **(A, E)** CT-based clinical-RN1; **(B, F)** CTE-based clinical-RN1; **(C, G)** CT-based clinical-RN2; **(D, H)** CTE-based clinical-RN2. For clinical-RNs (the first row), a straight line was drawn to determine the points for each feature (for example, features including size, type, location, operation methods, sex and radscore for **(A)**. The final “Total points” were calculated by summing the score of each point represented for each feature. After the total points is calculated, a vertical line is drawn at the value corresponding to the line “Total Points”, and the value corresponding to the line “Risk” represents the probability of MBL. The corresponding calibration curves of the clinical-RNs in the training set (left) and validation set (right) were displayed in the second row. The nomogram-predicted probabilities were shown in x axis and the actual probability was represented on the y axis. The closer the two dotted line, the better the prediction.

The radscores of different RNs were calculated by using the formula listed in the Electronic **Supplementary Material**. In the CT-based clinical-RN1 model, multivariable LR analyses showed that radscore, tumor type, tumor location, and sex (odds ratio [OR]1 = 2.109, OR2 = 0.833, OR3 = 1.224, OR4 = 1.958, *P* < 0.05) were important predictors of the MBL of pelvic and sacral tumors, of which radscore was the most important. In the CTE-based clinical-RN1, radscore, tumor type, and tumor location (OR1 = 2.181, OR2 = 0.833, OR3 = 1.352, *P* < 0.05) were significant predictors of MBL. In this model, radscore was the most important factor, followed by tumor location. In the CT-based clinical-RN2 model, radscore (OR = 4.706, *P* < 0.01) and sex (OR = 2.13, *P* < 0.05) were significant predictors of MBL. In the CTE-based clinical-RN2 model, however, only radscore (OR = 3.844, *P* < 0.01) was important independent factor (**Figure 3** and **Table 3**).

**Table 3:** Multivariable logistic regression analyses.

Variable	CT			CTE		
	Coefficient	OR (95% CI)	*P*	Coefficient	OR (95% CI)	*P*
Clinical-RN1						
Intercept	-2.3220	–	0.0002	-2.2204	–	0.0014
Radscore	0.7462	2.109 (1.586, 2.804)	<0.0001	0.7797	2.181 (1.518, 3.133)	<0.0001
Tumor size	0.0130	1.013 (0.943, 1.089)	0.7231	-0.0500	1.051 (0.963, 1.147)	0.2631
Tumor type	-0.1239	0.833 (0.798, 0.978)	0.0171	-0.1827	0.833 (0.742, 0.935)	0.0020
Tumor location	0.2021	1.224 (1.01, 1.484)	0.0397	0.3016	1.352 (1.099, 1.663)	0.0043
Operation method	0.2433	1.275 (0.826, 1.968)	0.2719	0.2652	1.304 (0.812, 2.092)	0.2717
Sex	0.6719	1.958 (1.178, 3.255)	0.0096			
Clinical-RN2						
Intercept	-0.5277	–	0.4182	-1.5259	–	0.1384
Radscore	1.5489	4.706 (1.96, 11.304)	0.0005	1.3465	3.844 (1.994, 7.41)	0.0001
Tumor size	0.0068	1.007 (0.895, 1.132)	0.9090	0.0353	1.036 (0.907, 1.183)	0.6017
Tumor location				0.3148	1.37 (0.964, 1.947)	0.0794
Sex	0.7559	2.13 (1.038,4.369)	0.0392			

OR, odds ratio; CI, confidence interval.

**Figure 4:**
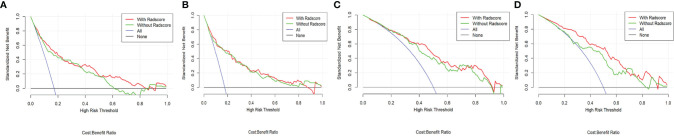
Decision curve of different clinical-RNs. **(A)**, CT-based clinical-RN1; **(B)**, CTE-based clinical-RN1; **(C)**, CT-based clinical-RN2; **(D)**, CTE-based clinical-RN2. The green line represents the clinical model. The red line represents the clinical-RN. Decision curve analysis showed that clinical-RNs achieved more clinical utility than clinical model.

Good agreement was found between the nomogram prediction and actual observation of the MBL of pelvic and sacral tumors, as shown in the calibration plots. The Hosmer–Lemeshow test results were not significant (*P* > 0.05), indicating a good fit. DCA showed that clinical-RN had more benefit than clinical nomograms (**Figure 4**).

## Discussion

In this study, we found that radscore, tumor type, tumor location, and sex were the significant predictors of the MBL of pelvic and sacral tumors. The clinical-DNN and clinical-RN performed better than DNN and RN. Both clinical-DNN and clinical-RN could be powerful tools for preoperatively predicting the MBL of patients with pelvic and sacral tumors, but the clinical-DNN model based on CT features was superior. Our model could help clinicians develop individualized treatment plans, prepare blood products in advance, and reduce the risk of surgical failure.

Given the small number of pelvic and sacral tumors, previous studies on intraoperative blood loss in these tumors were limited and had small sample sizes. In addition, these studies failed to reach a consensus on the factors that affect the amount of blood lost during surgery. Tang et al. (1, 10) found that gender, tumor blood supply, tumor location, tumor volume, aorta occlusion, surgical approach, reconstruction, and operative time are associated with large blood loss from sacral and pelvic tumors, of which tumor location was the most important. In our study, multivariable LR analyses showed that radscore, tumor type, tumor location, and sex were the important predictors of the MBL of pelvic and sacral tumors, with radscore being the most important. Radscore can reflect the heterogeneity of different tumors, which has been proven to be an important predictor of tumor recurrence, metastasis, and classification (2, 27–29). Our results demonstrated that the proportion of males in the MBL group was high, and sex was an independent predictor of MBL. Tang et al. (10) also found that male gender is associated with large blood loss in sacral tumors. In our study, we divided the tumor types into 10 categories in accordance with the pathological results and found that MBL was more likely to occur in chondrosarcoma and osteosarcoma. In general, pelvic and sacral tumors are often large when detected and are likely to invade adjacent blood vessels and organs, resulting in increased surgical difficulty and MBL risk. Our results showed that the proportion of tumors in zone IV was the largest, and MBL was more likely to occur when multiple sites were involved. Although surgeon and surgical method were significantly associated with MBL, they were not independent predictors of MBL. In addition, patients with aortic balloon occlusion had significantly more blood loss than patients without occlusion, which may be related to the surgeon’s bias in the use of aortic occlusion in patients with a tendency for MBL. Contrary to expectations, embolism was not a significant risk factor of MBL, which might also be explained by the selection bias in our data.

Considering our imbalanced data, we grouped the data in two ways. The SMOTE method was used in the randomization method for the training set, which was beneficial for feature selection and model building (30, 31). Our results demonstrated that the clinical model had a relatively higher performance than individual RN or DNN in the validation set, indicating that clinical indicators are important for the prediction of intraoperative MBL. Gao et al. (7) constructed an effective clinical model for predicting intraoperative blood loss for metastatic spinal tumors and found a predictive and actual correlation coefficient of only 0.606 in the validation group. In this study, we built multiple fusion models by combining clinical data with RN or DNN. Our DNN model had 3 hidden layers, which can simplify problems and improve efficiency (32). Some previous studies have also shown that deep learning performs better than RN in tumor classification and prognosis (33, 34). In this study, we found that both clinical-RN1 and clinical-RN2 had better performance than individual RN in terms of CT or CTE features. Clinical-DNN based on CT features performed the best among all the models. Our combined models, which were based on radiomics or DNN methods, could provide a simple and accurate way to predict intraoperative MBL and help clinicians develop personalized treatment plans in advance.

Our study has certain limitations. First, all images were collected from a single center over the past decade or so. Patients who did not receive preoperative CT examination were excluded, which may lead to selective bias. Although we strictly screened the included large sample data, a multicenter study is beneficial to future research. Second, a certain imbalance existed between the number of MBL cases and that of non-MBL cases. Although we adopted two modeling methods and found no significant difference between RN1 and RN2, the sensitivity and specificity of our models were still affected to some extent. Third, we used a semi-automatic method to sketch all lesions, which was time-consuming. We will consider using an automatic segmentation method in further studies.

In conclusion, DNN and RN, especially when combined with clinical features, showed good performance in predicting the MBL of pelvic and sacral tumors. The clinical-DNN and clinical-RN can be powerful tools for preoperatively predicting the MBL of patients with pelvic and sacral tumors and may reduce the risk of surgical failure due to the preoperative misjudgment of blood loss.

## Data Availability Statement

The original contributions presented in the study are included in the article/**Supplementary Material**. Further inquiries can be directed to the corresponding author.

## Ethics Statement

This study was approved by the local ethics committee of our hospital, and written informed consent was waived.

## Author Contributions

Conceptualization: PY and NH. Data acquisition: PY and CS Formal analysis: PY and SW. Investigation: PY. Methodology: PY, SW, and CS. Project administration: NH and LC. Resources: PY and CS. Software: SW and CS. Supervision: NH and LC. Validation: SW and PY. Visualization: PY and NH. Roles/Writing - original draft: PY. Manuscript editing & review: PY and NH. All authors contributed to the article and approved the submitted version.

## Funding

Project (RDY2020-08) supported by Peking University People’s Hospital Scientific Research Development Funds. National Natural Science Foundation of China, No.82001764.

## Conflict of Interest

Author SW was employed by company GE healthcare.

The remaining authors declare that the research was conducted in the absence of any commercial or financial relationships that could be construed as a potential conflict of interest.

## Publisher’s Note

All claims expressed in this article are solely those of the authors and do not necessarily represent those of their affiliated organizations, or those of the publisher, the editors and the reviewers. Any product that may be evaluated in this article, or claim that may be made by its manufacturer, is not guaranteed or endorsed by the publisher.

## Supplementary Material

Supplementary Figure 1 DNN diagram. The number of hidden layers in DNN model is 3, and the number of nodes in each layer is 4, 3 and 2 respectively.

## Abbreviations

MBL, massive blood loss; DNN, deep neural network; RN, radiomics nomogram; mRMR, minimum redundancy maximum relevance; LASSO, least absolute shrinkage and selection operator; SMOTE, synthetic minority oversampling technique; LR, logistic regression; AUC, area under the receiver operating characteristic curve; ACC, accuracy.
