# Supplementary figures and images for: Clinical-Deep Neural Network and Clinical-Radiomics Nomograms for Predicting the Intraoperative Massive Blood Loss of Pelvic and Sacral Tumors
Source: Front Oncol. 2021 Oct 25;11:752672. doi: 10.3389/fonc.2021.752672 (PMC8574215; doi:10.3389/fonc.2021.752672)

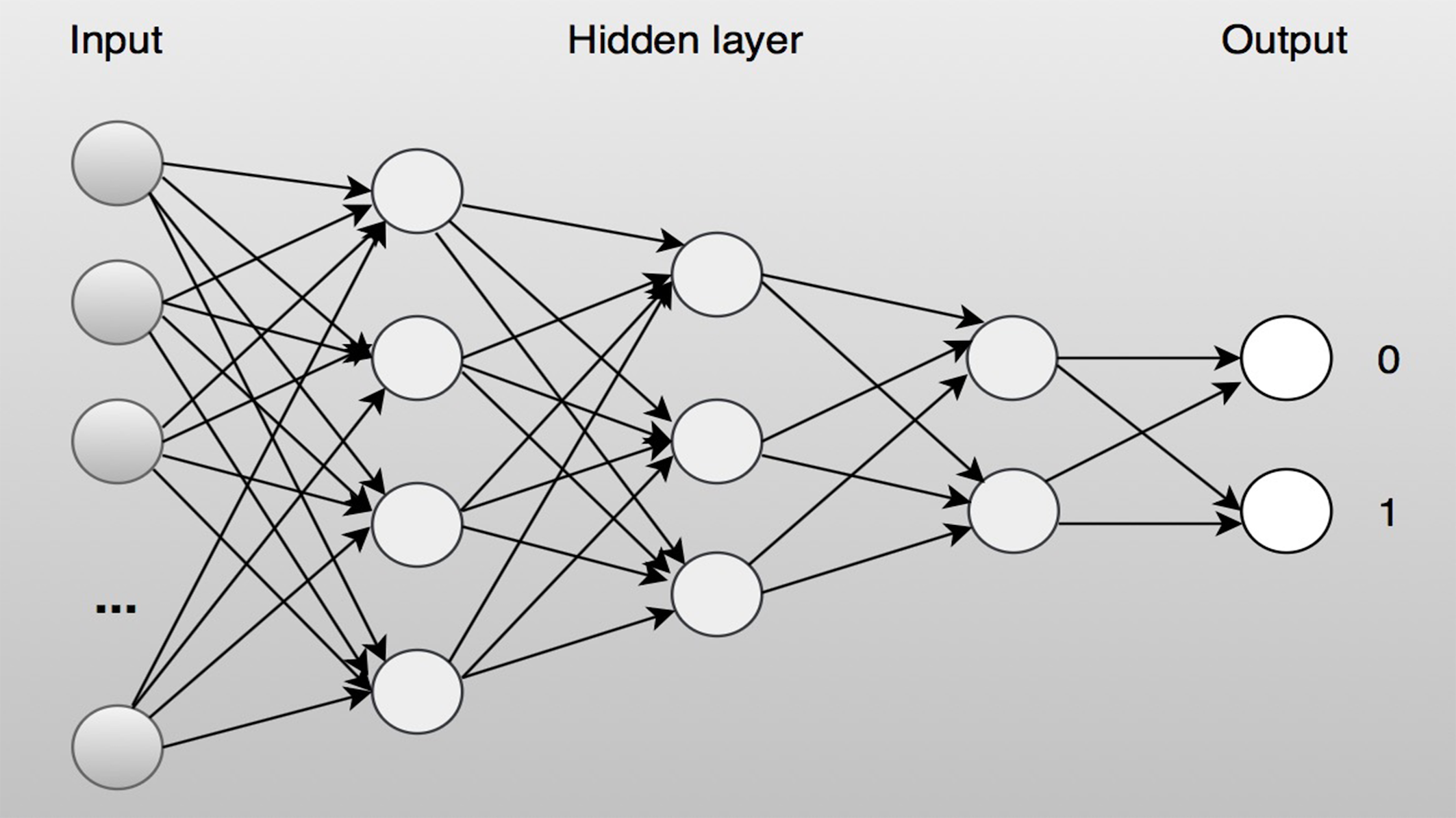

Supplement: Supplementary Figure 1 — DNN diagram. The number of hidden layers in DNN model is 3, and the number of nodes in each layer is 4, 3 and 2 respectively. [file Image_1.tif]
