# Supplementary material for: Clinical-Deep Neural Network and Clinical-Radiomics Nomograms for Predicting the Intraoperative Massive Blood Loss of Pelvic and Sacral Tumors
Source: Front Oncol. 2021 Oct 25;11:752672. doi: 10.3389/fonc.2021.752672 (PMC8574215; doi:10.3389/fonc.2021.752672)
Supplement: Supplementary file 2 [file Table_1.docx]

Supplemental Table 1 Clinical characteristics of the randomized training and validation groups

| Variable | CT | | CTE | |  |  |
| --- | --- | --- | --- | --- | --- | --- |
|  | Training | Validation | Training | Validation | *t/χ^2^*/Z value | P value |
| Sex |  |  |  |  |  |  |
| Female | 253(44.62%) | 112(46.09%) | 207(46.00%) | 83(43.01%) | 0.148^a^ (0.489^b^) | 0.700^a^ (0.484^b^) |
| Male | 314(55.38%) | 131(53.91%) | 243(54.00%) | 110(56.99%) |  |  |
| Age (years) | 42.43±16.94 | 42.61±18.05 | 44.50(28.00, 57.00) | 43.00(30.00, 55.00) | 0.142^a^ (-0.280^b^) | 0.887^a^ (0.779^b^) |
| Tumor type |  |  |  |  |  |  |
| Metastatic tumor | 97(17.11%) | 41(16.87%) | 75(16.67%) | 30(15.54%) | 6.040^a^ (6.781^b^) | 0.736^a^ (0.660^b^) |
| Chordoma | 57(10.05%) | 27(11.11%) | 46(10.22%) | 25(12.95%) |  |  |
| Giant cell tumor | 83(14.64%) | 32(13.17%) | 64(14.22%) | 27(13.99%) |  |  |
| Osteosarcoma | 65(11.46%) | 33(13.58%) | 62(13.78%) | 21(10.88%) |  |  |
| Chondrosarcoma | 92(16.23%) | 31(12.76%) | 65(14.44%) | 35(18.13%) |  |  |
| Schwannoma | 35(6.17%) | 14(5.76%) | 23(5.11%) | 13(6.74%) |  |  |
| Neurofibroma | 33(5.82%) | 12(4.94%) | 25(5.56%) | 12(6.22%) |  |  |
| Ewing’s sarcoma | 44(7.76%) | 16(6.58%) | 31(6.89%) | 13(6.74%) |  |  |
| Multiple myeloma | 12(2.12%) | 6(2.47%) | 9(2.00%) | 1(0.52%) |  |  |
| Others | 49(8.64%) | 31(12.76%) | 50(11.11%) | 16(8.29%) |  |  |
| Tumor size (cm) | 9.63±4.05 | 9.48±3.86 | 9.00(6.90, 12.00) | 8.80(6.50, 12.03) | -0.460^a^ (-0.663^b^) | 0.646^a^ (0.507^b^) |
| Tumor location |  |  |  |  |  |  |
| I | 78(13.76%) | 45(18.52%) | 72(16.00%) | 26(13.47%) | 3.193^a^ (4.075^b^) | 0.526^a^ (0.396^b^) |
| II | 37(6.53%) | 16(6.58%) | 32(7.11%) | 9(4.66%) |  |  |
| III | 34(6.00%) | 15(6.17%) | 23(5.11%) | 14(7.25%) |  |  |
| IV | 297(52.38%) | 117(48.15%) | 216(48.00%) | 103(53.37%) |  |  |
| Multiple locations | 121(21.34%) | 50(20.58%) | 107(23.78%) | 41(21.24%) |  |  |
| Neoadjuvant chemoradiotherapy |  |  |  |  |  |  |
| No | 404(71.25%) | 177(72.84%) | 319(70.89%) | 139(72.02%) | 0.211^a^ (0.084^b^) | 0.646^a^ (0.771^b^) |
| Yes | 163(28.75%) | 66(27.16%) | 131(29.11%) | 54(27.98%) |  |  |
| Embolism |  |  |  |  |  |  |
| No | 304(53.62%) | 142(58.44%) | 252(56.00%) | 110(56.99%) | 1.598^a^ (0.054^b^) | 0.206^a^ (0.816^b^) |
| Yes | 263(46.38%) | 101(41.56%) | 198(44.00%) | 83(43.01%) |  |  |
| Surgeon |  |  |  |  |  |  |
| Surgeon 1 | 38(6.70%) | 12(4.94%) | 31(6.89%) | 11(5.70%) | 12.866^a^ (3.250^b^) | 0.231^a^ (0.975^b^) |
| Surgeon 2 | 251(44.27%) | 101(41.56%) | 194(43.11%) | 91(47.15%) |  |  |
| Surgeon 3 | 48(8.47%) | 22(9.05%) | 43(9.56%) | 16(8.29%) |  |  |
| Surgeon 4 | 75(13.23%) | 34(13.99%) | 60(13.33%) | 27(13.99%) |  |  |
| Surgeon 5 | 41(7.23%) | 22(9.05%) | 29(6.44%) | 13(6.74%) |  |  |
| Surgeon 6 | 10(1.76%) | 5(2.06%) | 9(2.00%) | 3(1.55%) |  |  |
| Surgeon 7 | 5(0.88%) | 10(4.12%) | 8(1.78%) | 4(2.07%) |  |  |
| Surgeon 8 | 12(2.12%) | 4(1.65%) | 11(2.44%) | 2(1.04%) |  |  |
| Surgeon 9 | 40(7.05%) | 14(5.76%) | 33(7.33%) | 13(6.74%) |  |  |
| Surgeon 10 | 33(5.82%) | 15(6.17%) | 21(4.67%) | 10(5.18%) |  |  |
| Surgeon 11 | 14(2.47%) | 4(1.65%) | 11(2.44%) | 3(1.55%) |  |  |
| Operation methods |  |  |  |  |  |  |
| Method1 | 312(55.03%) | 121(49.79%) | 234(52.00%) | 103(53.37%) | 2.944^a^ (0.935^b^) | 0.229^a^ (0.627^b^) |
| Methods2 | 234(41.27%) | 108(44.44%) | 197(43.78%) | 79(40.93%) |  |  |
| Methods3 | 21(3.70%) | 14(5.76%) | 19(4.22%) | 11(5.70%) |  |  |
| Surgical approaches |  |  |  |  |  |  |
| Approach1 | 538(94.89%) | 226(93.00%) | 422(93.78%) | 179(92.75%) | 1.124^a^ (0.235^b^) | 0.289^a^ (0.627^b^) |
| Approach2 | 29(5.11%) | 17(7.00%) | 28(6.22%) | 14(7.25%) |  |  |
| Balloon occlusion |  |  |  |  |  |  |
| No | 133(23.46%) | 72(29.63%) | 100(22.22%) | 54(27.98%) | 3.429^a^ (2.458^b^) | 0.064^a^ (0.117^b^) |
| Yes | 434(76.54%) | 171(70.37%) | 350(77.78%) | 139(72.02%) |  |  |

Note: Operation methods, Method1 = resection of sacral tumors and internal fixation, Methods2 = resection of left or right pelvic tumors plus artificial hemipeleal replacement, Methods3 = resection and internal fixation of sacral and pelvic tumors. Approaches, Approach1 = posterior, Approach2 = combined anterior and posterior. a, CT. b, CTE.
